# Thalamocortical Connectivity in Experimentally-Induced Migraine Attacks: A Pilot Study

**DOI:** 10.3390/brainsci11020165

**Published:** 2021-01-27

**Authors:** Daniele Martinelli, Gloria Castellazzi, Roberto De Icco, Ana Bacila, Marta Allena, Arianna Faggioli, Grazia Sances, Anna Pichiecchio, David Borsook, Claudia A. M. Gandini Wheeler-Kingshott, Cristina Tassorelli

**Affiliations:** 1 Headache Science Center, IRCCS Mondino Foundation, 27100 Pavia, Italy; roberto.deicco@mondino.it (R.D.I.); marta.allena@mondino.it (M.A.); grazia.sances@mondino.it (G.S.); cristina.tassorelli@unipv.it (C.T.); 2 Department of Brain and Behavioral Sciences, University of Pavia, 27100 Pavia, Italy; anna.pichiecchio@mondino.it (A.P.); c.wheeler-kingshott@ucl.ac.uk (C.A.M.G.W.-K.); 3 NMR Research Unit Queen Square MS Centre, Department of Neuroinflammation, UCL Institute of Neurology, Faculty of Brain Sciences, London WC1N3BG, UK; gloria.castellazzi@mondino.it; 4 Department of Electrical Computer and Biomedical Engineering, University of Pavia, 27100 Pavia, Italy; 5 IRCCS Mondino Foundation, 27100 Pavia, Italy; 6 Center of Advance Imaging and Radiomics, IRCCS Mondino Foundation, 27100 Pavia, Italy; ana.bacila@mondino.it (A.B.); arianna.faccioli@mondino.it (A.F.); 7 Centre for Pain and The Brain Boston Children’s Hospital and Massachussetts General Hospital (MGH) Harvard Medical School, Boston, MA 02115, USA; dborsook@me.com

**Keywords:** NTG-induction, fMRI, thalamus, SCA, WCA, task-free functional MRI, migraine

## Abstract

In this study we used nitroglycerin (NTG)-induced migraine attacks as a translational human disease model. Static and dynamic functional connectivity (FC) analyses were applied to study the associated functional brain changes. A spontaneous migraine-like attack was induced in five episodic migraine (EM) patients using a NTG challenge. Four task-free functional magnetic resonance imaging (fMRI) scans were acquired over the study: baseline, prodromal, full-blown, and recovery. Seed-based correlation analysis (SCA) was applied to fMRI data to assess static FC changes between the thalamus and the rest of the brain. Wavelet coherence analysis (WCA) was applied to test time-varying phase-coherence changes between the thalamus and salience networks (SNs). SCA results showed significantly FC changes between the right thalamus and areas involved in the pain circuits (insula, pons, cerebellum) during the prodromal phase, reaching its maximal alteration during the full-blown phase. WCA showed instead a loss of synchronisation between thalami and SN, mainly occurring during the prodrome and full-blown phases. These findings further support the idea that a temporal change in thalamic function occurs over the experimentally induced phases of NTG-induced headache in migraine patients. Correlation of FC changes with true clinical phases in spontaneous migraine would validate the utility of this model.

## 1. Introduction

Episodic migraine, a disease with a complex pathophysiology, has been a challenge to study because of the nature of the timing of the disease [1,2]. The use of chemically induced (CI) approaches to precipitate a headache in patients with migraine allows for a controlled method for evaluating pathophysiological changes in such patients. The use of CI headaches in migraine patients has been proposed to model the true migraine attack. However, evidence for this is still lacking. One approach is to compare fMRI changes in brain systems following CI headaches across time with those brain changes in spontaneous migraine.

Advanced neuroimaging techniques, including resting state functional magnetic resonance imaging (fMRI), which allows to assess changes in static and dynamic functional connectivity (FC) when the brain is at “rest”, have emerged as a leading non-invasive candidate to investigate the disease-induced neural dysfunction associated with migraine. Several independent studies reported the evidence of increased FC within the pain-processing network, coupled with decreased FC in the circuitries involved in pain modulation [3] and more specifically in the salience network (SN) [4,5]. Further studies showed also significant FC changes in large-scale networks such as the default mode network (DMN) [6,7] and executive control network (ECN) [8]. The overall output of these studies suggests that migraine attacks represent a disorder of the brain sensory processing. Under this condition, several brain regions cyclically modify their functional coupling producing the complex phenomenology experienced by the patient [9]. In this framework, the thalamus emerges as a pivotal point of relay in the migraine cycle both from a structural perspective, for the ascending nociceptive information, via the trigemino-thalamocortical pathway from lower brain areas to various cortical regions, and from a functional perspective [10], when considering the abnormal thalamocortical connection dynamics in migraine ictal and inter-ictal phases, [11], specifically accounting for the alteration within the salience network (SN). Furthermore, the thalamus is also a site of central sensitisation in migraine pathophysiology [12].

As part of the analysis methods available to process static fMRI data, the seed-based correlation approach (SCA) allows to detect the full connectivity profile of a brain area of interest (labelled as *seed* area), showing all the brain regions that present significant functional coupling (i.e., synchronous time course fluctuations) with the seed in the connectivity map. A fundamental example of this approach, where clinical and literature data drove the selection of a particular seed, has been proved by Schulte and May [13]. Their studies demonstrated that the posterior part of the hypothalamus shows an altered FC with the spinal trigeminal nuclei and the region of the dorsal rostral pons, according to the state of the migraine cycle, thus likely becoming the leading candidates for attack generation [14]. 

Widening the perspective, other methods involving the time-frequency analysis have been developed to characterize temporal varying functional connectivity between different regions/networks and applied to psychiatric and neurological pathologies such as multiple sclerosis [15]. New approaches involving wavelet decomposition, such as the wavelet-coherence analysis (WCA) [16], represent powerful methods to assess transient or non-stationary processes to get insights into the brain temporal dynamics. In particular, WCA has been applied to evaluate large resting state networks’ altered dynamic in patients affected by an autism spectrum disorder [17], but only rarely to study the dynamic activity of migraineurs’ brain. Wang et al. [18], for example, used it to compare chronic migraineurs in the interictal phase and matched healthy controls s highlighting robust and significant differences (reduction) in temporal dynamics FC between two regions of interest (ROI) pairs involving left medial-orbitofrontal–left posterior-cingulate and left medial-orbitofrontal–left inferior-temporal regions. These areas are core parts of the default mode network (DMN), executive control, sensorimotor and salience resting state networks. To our knowledge this approach has never been applied to study an acute migraine attack: a condition hardly ever spontaneously evaluated with a standardised protocol.

From an experimental perspective, a peculiar feature of migraine without aura is the possibility of provoking attacks with specific triggers. Among the various triggers, nitroglycerin (NTG) provocation in humans and animals has been extensively studied as a model of migraine. A large body of evidence collected over the years using this model has witnessed its usefulness in the study of migraine [19]. A striking similarity exists indeed between spontaneous migraine attacks and NTG-induced headache attacks in migraineurs, specifically without aura, [20] thus warranting the use of this paradigm as a potential model for the study of migraine in both animals and humans with standardised and reproductible protocols. In one of the first studies evaluating NTG changes with fMRI, [21], changes in brain regions including the thalamus were observed during different ‘putative’ phases of the NTG induced headache. While contributing to approaches of experimentally induced migraine, the last study did not provide evidence of brain changes that were paralleled in the natural state of a spontaneous condition.

Here we provide a further study of the NTG-induced migraine attack model using task-free advanced MRI techniques, in order to depict the static and dynamic changes of the ictal and inter-ictal phase. This type of acquisition and analysis of fMRI data can be applied in the future to compare this model with brain changes in spontaneous episodic migraine attacks as a validation step. To this end, fMRI images were processed SCA and WCA approaches to assess whether and which functional brain pathways presented a consistent altered FC during all the different phases of an NTG-triggered headache attack in episodic migraineurs considering the thalamus as seed for the reasons noted above.

## 2. Materials and Methods

### 2.1. Subjects

Subjects with episodic migraine (EM) were consecutively recruited over one year at the Headache Science Centre (a tertiary referral centre) of the IRCCS Mondino Foundation in Pavia, Italy. Inclusion criteria were: age between 18–60 years; diagnosis of EM without aura developed before the age of 50 with a moderate-to-high intensity of attacks; no current prophylactic treatment for migraine prevention; historical antimigraine efficacy of non-steroidal anti-inflammatory drugs. Exclusion criteria were: overuse of acute medication for headache; diagnosis of cluster headache; a diagnosis of tension type headache with a frequency of more than 2 days per month; any chronic pain condition or disorders other than migraine; major psychiatric disorders such as depression, bipolar affective disorder and schizophrenia; cardiovascular diseases that contraindicated the use of NTG; blood pressure hypotension, closed angle glaucoma, anaemia; pregnant women or breast feeding women; frequent use of benzodiazepines; any neuroradiological pathological findings, different from those related to the disease, at a previous MRI scan of the head. The study was approved by the local ethics committee, it was registered online in the ClinicalTrial.gov database (NCT04503083) and all subjects provided written informed consent before enrolment in the study. The acquisitions were carried out in the first semester of 2019.

### 2.2. Clinical Assessment

All subjects underwent clinical evaluation to assess their health status by an expert neurologist. Migraine diagnosis was made according to the International Classification of Headache Disorders, 3rd edition version ICHD-III [22]. Furthermore, headache signs and symptoms were clinically evaluated and recorded for each subject during their progression through the protocol. All acquired clinical data were tested for significant differences across the migraine attack phases using the χ^2^ test.

### 2.3. MRI Acquisition

**Figure 1:**
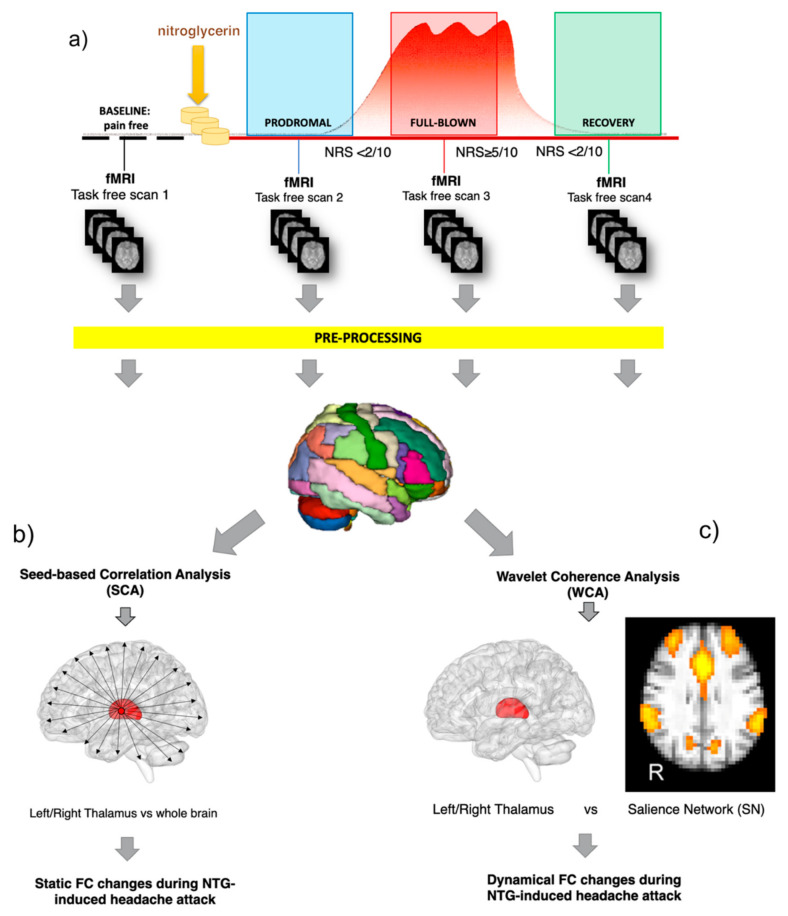
Workflow of the study. (**a**) Each subject underwent four fMRI scans in a day, matched with the different attack phases (baseline at a pain-free condition, prodromal, full blown, recovery) before and after the nitroglycerin (NTG) administration. fMRI data were first pre-processed and then treated with two different methodological approaches: (**b**) seed-based correlation analysis (SCA) and (**c**) the wavelet coherence analysis (WCA). SCA was applied on fMRI to assess the static functional connectivity (FC) changes between the thalamus and the rest of the brain. WCA was applied to test the time varying phase-coherence (dynamic) changes between the thalamus and the salience network (SN).

All subjects underwent MRI examination four times in a day (see Figure 1a) using a 3T Siemens Skyra scanner (Siemens, Erlangen, Germany) with a 32-channel head coil. The total acquisition time (AT) of each MRI scan was about 18 min. The MRI acquisition protocol, repeated during the study design as explained below, included: (1) fMRI: multi-band T2*-weighted Gradient Echo echo-planar (GRE-EPI) sequence (TR/TE = 3010/20 ms; MB = 2, voxel size = 2.5 mm^3^ isotropic, FOV = 224 mm^2^, 60 slices, 200 volumes; (2) a high-resolution 3D sagittal T1-weighted (3DT1) scan (MPRAGE sequence: TR/TE/TI = 2300/2.96/900 ms, flip angle = 9°, FOV = 256 mm^2^, 156 slices, voxel size = 1 mm^3^ isotropic) was also acquired for anatomical reference. During the fMRI acquisition, participants were asked to keep their eyes closed and neither to think about anything in particular, nor to sleep, as to acquire a resting state condition which can only be considered represented in the baseline pain free scan. Light inside the scanner room was switched off in order to reduce the photophobia experience. 

### 2.4. NTG-Induction Paradigm

The study design is shown in Figure 1. Before the NTG challenge visit, subjects with EM were informed about the possibility that the procedure might induce a spontaneous-like attack and were instructed to recognise the different features of the prodromal and full-blown phase of the attack. On the day of the challenge, the enrolled EM subjects underwent both clinical and imaging assessments within the NTG-induction procedure, which included detailed recording of symptoms at serial (15 min.) intervals and 4 MRI sessions, all performed on the same day at specific time points during the evolution of the NTG-induced attack. The evaluation of the pain intensity was performed with a Numeric Rating Scale (NRS) scoring from 0 to 10 (mild: 1–3; moderate: 4–6; severe: 7–10). In detail, the study protocol consisted in six consecutive steps as follows: (1) Scan 1—Baseline: acquired in a pain-free condition during interictal period at least 72 h resolution of any prior migraine attack; (2) NTG administration: supervised administration of 3 sublingual tablets of 0.3 mg and monitoring of vital signs every 15 min during the first hour; (3) Scan 2—Prodrome: this was acquired at the occurrence of at least two of the symptoms typical of the prodromal phase of an NTG-triggered migraine-like headache out of a list of possible features.; (4) Scan 3—Full Blown: acquired during the migraine-like full-blown headache attack (i.e., NRS ≥ 5); (5) Recovery: after the full-blown scan, the patient is treated with an effective non-steroidal anti-inflammatory drug (NSAID). (6) Scan 4—Recovery: acquired during the postdrome phase (i.e., the recovery phase after the headache resolution, NRS ≤ 1).

*fMRI pre-processing:* All fMRI data were pre-processed using FSL (FMRIB Software Library, version 5.0.9, http://www.fmrib.ox.ac.uk/fsl/) and Matlab (v. R2019b, The Mathworks, Inc., Natick, MA) as described in Castellazzi et al. [23]. Individual pre-processing steps consisted of motion correction, brain extraction, spatial smoothing using a Gaussian kernel of a full-width-at-half-maximum (FWHM) of 5 mm, and high-pass temporal filtering equivalent to 150 s (0.007 Hz). For each subject, individual fMRI volumes were linearly registered to the corresponding structural 3DT1 scan and subsequently to standard space (MNI152) using the NiftyReg toolbox (http://niftyre.g.sf.net). To reduce the nuisance effects of non-neuronal BOLD fluctuations, the white matter (WM) and the cerebrospinal fluid (CSF) signals were regressed out of fMRI data.

*Cortical and subcortical ROI definition:* For each subject, pre-processed fMRI images were parcellated into 166 distinct areas obtained by unifying in a single atlas both the 32 regions of the SUIT atlas [24] and the geodesic information flows (GIF) atlas [25]. Finally, eight more regions of interest for small volume correction were generated via the FSL Toolbox using the coordinates of previously reported activations within the respective brain areas as centre of spheres. Sphere diameters varied according to the anatomical properties of the brain region the respective region of interest was located in, as summarised in Table 1. Given the demonstrated role of the thalamus as a key hub in pain perception and sensory modulation via thalamocortical pathways, [1,26,27,28], the left and right thalamic areas were selected as seeds for the SCA and WCA.

*Seed-based correlation analysis (SCA):* For each subject, the pre-processed parcellated fMRI was treated with a SCA approach, implemented as a voxel-wise multiple regression analysis (see Figure 1). Specifically, for each seed ROI (i.e., left thalamus, right thalamus), the average fMRI time-course was extracted and used as *reference* for the cross-correlation analysis (i.e., the SCA). For each seed ROI, the result of this operation was a set of four SCA maps, labelled SCA-baseline, SCA-prodrome, SCA-full-blown and SCA-recovery map, where the intensity value of each voxel reflects the Pearson correlation coefficient between the voxel time-course and the reference time-course. SCA maps were then used for statistical testing to assess the group mean effect (ME) using a non-parametric permutation test, referred to as “dual regression” technique using 5000 permutations and age, gender and disease duration as additional covariates in the analysis [32]. The resulting statistical maps (*tstat_ME_*) were corrected for threshold-free cluster enhancement (TFCE) and multiple comparisons using the family-wise error (FWE) procedure. A statistical threshold of *p* < 0.05 was considered significant.

*SCA map comparison:* For an objective assessment of the changes of the SCA maps across the four scans of the fMRI protocol, we used the individual subject’s mean FC maps, as output from the dual regression step, to obtain values of the FC magnitude for each voxel and for each SCA map, averaged across the group (i.e., mean FC value of non-zero voxels per scan across subjects) over the areas identified by the corresponding *tstat_ME_* maps. We then calculated a “relative change in FC” (*rcFC*) index by normalising each SCA map for baseline FC values:

*rcFC_x_* = [(*meanFC_x_* − *meanFC_baseline_*)/*meanFC_baseline_*] (1)

where *x* represents the scan of interest for the evaluation of the *rcFC* index (i.e., prodrome, full-blown, recovery). For each seed area, calculated rcFC values among the phases (i.e., prodrome, full-blown and recovery) were statistically compared using the repeated measures ANOVA test with Bonferroni correction using SPSS (version 23.0, Chicago, IL, USA).

*ICA for salience network identification*: For each recruited subject, pre-processed fMRI images underwent group-ICA analysis using FSL to characterize the SN network. Specifically, pre-processed functional data, containing 200 time points (volumes) for each subject, were temporally concatenated across subjects to create a single 4-dimensional data set. The dataset was decomposed into independent components (ICs), with an automatic estimation of the number of components, which resulted in spatial maps used subsequently for assessing parameters’ time course over the four fMRI scans. Model order was estimated using the Laplace approximation to the Bayesian evidence for a probabilistic principal component model. Some of the ICs were identified as noise while others as resting state networks (RSNs), based on their frequency spectra and spatial patterns [33,34]. Based on previous literature, the SN spatial map was identified among the recognised RSNs [32].

**Figure 2:**
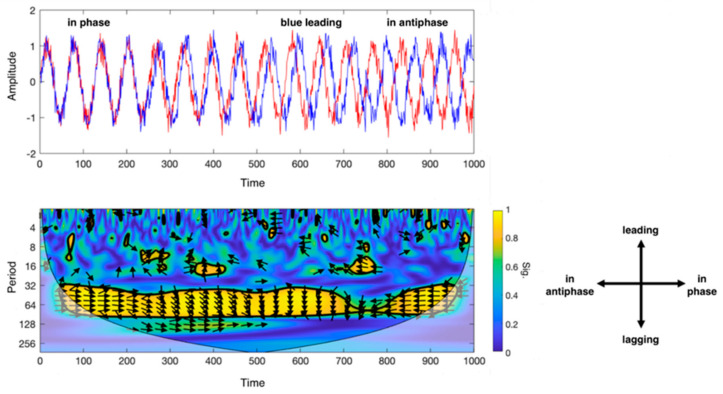
Description of the wavelet coherence analysis (WCA). Top left: example of phase coherence between two sinusoidal signals (in blue and red), from in-phase (on the left) to antiphase (on the right) condition during a defined set of acquisition time; bottom left: scalogram that outputs from WCA for the two sinusoidal signals. X-axis indicates the progression of time (which may be expressed in seconds, minutes or number of volumes if referred to MRI scans), y-axis the scale (in Fourier periods). The map threshold is set at 95% confidence by a thick black curve based on Monte Carlo tests. Regions outside the cone of influence appear in faded colour. Color scale reflects the statistical significance of the wavelet coherence between the two signals. The phase angle between ROI time series at a particular location in the time–frequency plane is indicated by an arrow. Bottom right: legend for the interpretation of the direction of the arrows (phase arrows). Phase arrows pointing: right—signals are in phase; left—signals are in antiphase; down—blue signal leading red signal by 90°; up—blues signal lagging red signal by 90°.

*Wavelet Coherence Analysis (WCA):* The WCA is based on the wavelet transform coherence (WTC), which allows to analyse the coherence and phase lag between two time series as a function of both time and frequency [35]. More specifically, WTC decomposes a time series in time-frequency domain by successively convolving the time series with the scaled and translated versions of a mother wavelet function [36]. In Torrence at al. [35], R^2^(s,τ) denotes the local correlation coefficients in time (τ) and wavelet scale (s), between two signals X and Y. The correlation coefficients are obtained by applying a wavelet cross-spectrum between the two signals and measuring the common power between the signals at various scales (s) and time (τ). The phase difference between X and Y is calculated with arg(R^2^(s,τ)). The results of these operations are combined in a scalogram, which is a map of wavelet coherence between the two signals (see Figure 2).

In this study, WCA was performed between the spontaneous oscillations of SN and bilateral thalami (see Figure 1), each taken individually. To this end, the SN spatial map, obtained from ICA, was then regressed into each subject’s space to give the SN time-course for each fMRI scan, for a total of four SN time-courses per subject. Then, the subject-specific SN time-courses were temporally concatenated to form a single time-course fMRI signal of 800 points. Similarly, for each subject, the average fMRI time-courses respectively from the left and right thalamic areas were extracted for each scan as described in the *seed-based correlation analysis* section and then temporally concatenated into single time-courses. The concatenated SN and thalamic signals were then averaged across subjects and the resulting time-courses were finally used as input for the WCA approach. WCA scalogram maps were produced using the Wavelet Coherence toolbox in Matlab (http://www.glaciology.net/wavelet-coherence) [37]. Specifically, WCA was performed using the complex Morlet wavelet as mother wavelet, enabling us to obtain phase information and therefore allowing visualization of directionality in the dynamics between signals (in-phase, leading, lagging, or anti-phase) [35]. The significance of the resulting coherence coefficients was tested against wavelet coherence of random red noise signals using Monte Carlo methods with 1000 surrogate data set pairs as described in Bernas et al. [17].

## 3. Results

### 3.1. Cohort Characteristics

A total of eight subjects were screened and seven were enrolled in the study. The seven subjects were exposed to the NTG trial, but two were excluded from final the analysis because either they did not develop a spontaneous-like attack or the MRI acquisition did not reach the quality standard required for the study. The final dataset consists of five subjects (3 males, 2 females, average age 33.4 ± 7.1 years) affected by EM without aura according to ICHD III criteria [22], mean onset of disease was at 13.2 years old (±4.2 years) and the average disease duration is 20.2 ± 7.2 years (see Table 2). Patients reported a migraine frequency of 4.4 ± 2.4 attacks per month. Two of them presented tension type headache as a comorbidity occurring an average of 1.2 ± 1 days per month. None of the patients was using any preventive migraine drugs and all subjects used a NSAID or triptan drug as abortive treatment with no current or past history of medication overuse. One patient reported comorbid generalised anxiety disorder which did not require daily pharmacological treatment. No other relevant medical history record was reported in the subjects analysed. All 5 patients who developed the NTG-induced headache attack not only fulfilled the ICHD III criteria for a migraine episode, but the clinical features were also directly comparable with the anamnestic characteristics described by each patient. No statistically significant differences were found when comparing each condition feature using the χ^2^ test (*p* > 0.05). The workflow and clinical characteristics of the attacks are summarised in Table 3.

### 3.2. Imaging

**Figure 3:**
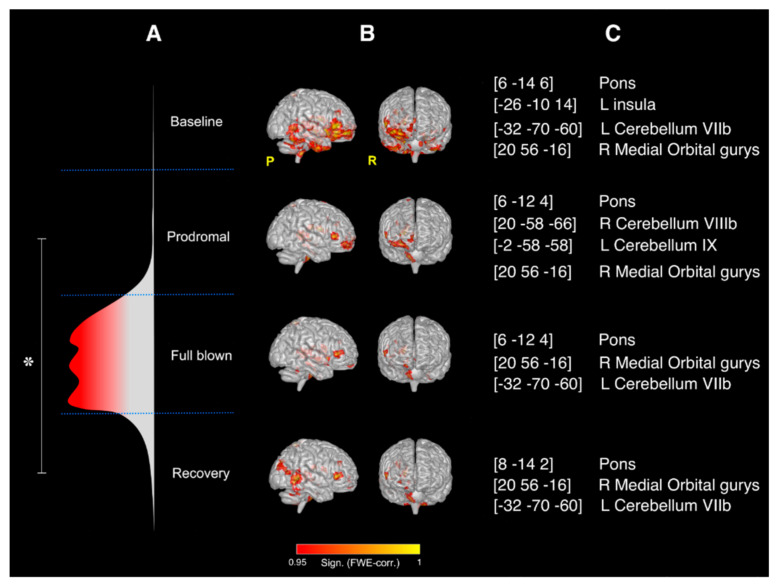
Results of the SCA analysis: right thalamus. (**A**) Flow chart of the study and representation of the different phases of the NTG-induced headache attack; overall significant changes (* FWE-corrected *p* < 0.05) in the rcFC values were only observed between the prodromal and the recovery SCA maps obtained using the right thalamus as seed area; (**B**) mean effect whole-brain intrinsic connectivity maps for seeds placed in the right thalamus during the four MRI scans of the study (i.e., the four phases of the NTG-induced headache attack) with relative significance (FWE corrected); (**C**) MNI coordinates (x,y,z) of the centroids and anatomical location of the major clusters of each mean effect map in (B).

Notwithstanding the small cohort, we found the following brain functional changes:

SCA results: Significant changes (*p* < 0.05) in the rcFC values were only observed between the prodromal and the recovery SCA maps obtained using the right thalamus as seed area (see Figure 3). No significant rcFC changes were observed among the four SCA maps when using the left thalamus as seed. The right thalamus mean effect connectivity maps during the experiment are shown in Figure 1b with the details of their anatomical location (expressed as centroids of the major clusters of each mean effect map) reported in Figure 1c. Overall, the right thalamus resulted significantly correlated (FWE-corrected *p* < 0.05,) with the pons and the contralateral posterior cerebellum (lobule VIIb) during the entire duration of the experiment. Moreover, compared to baseline, severe reductions in FC were observed during the prodromal phase of the migraine, reaching the maximal map reduction during the full-blown phase and partially recovering the initial condition (as at baseline) during the recovery. Full details of the areas involved in the SCA maps have been reported as supplementary information.

**Figure 4:**
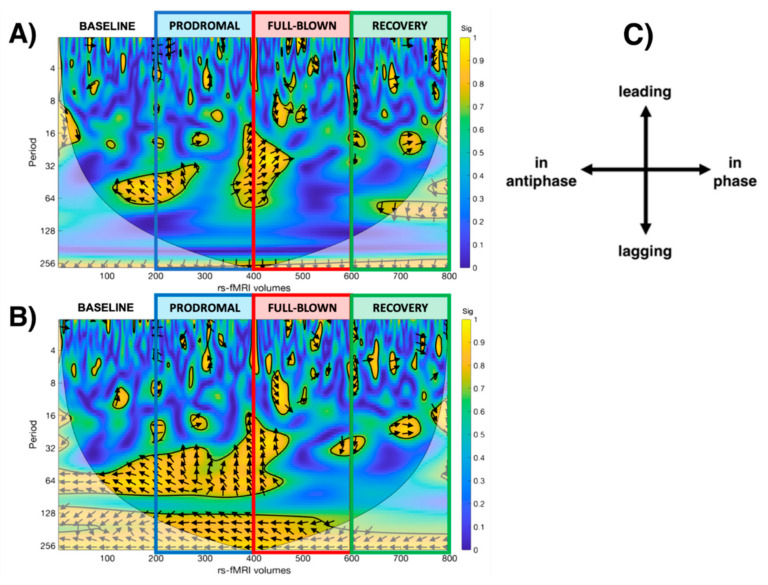
Scalogram representing the statistically significant WCA analysis results showing the dynamic correlation with the salience network during each phase of the study (baseline, prodromal, full-blown, recovery), considering the (**A**) left thalamus and the (**B**) right thalamus as a seed. (**C**) legend for the interpretation of the direction of the arrows (phase arrows).

WCA results: Both thalami and SN showed significant dynamical changes in their functional interaction during the experience of the NTG-induced headache attack (see Figure 4). At baseline, bilateral thalamic fMRI signals resulted in antiphase with SN, gradually losing phase synchronisation during the prodromal and full-blown phases, with the thalamic signals prevalently leading the SN. This loss of phase-coherence reached its peak during the full-blown phase. During the full-blown phase, both thalami showed only signs of phase lag with SN. During the recovery phase, the relationship between the thalami and the SN was in phase and gradually restored qualitatively resembling the baseline profile in the scalogram.

## 4. Discussion

This pilot study, which combined the experimental NTG human model of migraine with advanced combined fMRI approaches and novel mathematical algorithms, provides further support to the hypothesis that migraine is a dynamic functional disorder. The major finding of this pilot study was the characterisation of thalamocortical functional connectivity alterations during an NTG-induced migraine-like headache attack. The SCA analysis showed that the thalamus, the brainstem and cerebellar elements involved in pain circuits exhibited an altered functional coupling with one another, in particular during the prodromal and the full-blown phases of the migraine attack. Moreover, WCA analysis further highlighted changes and disruptions in the dynamic functional interactions between the different brain areas that are involved during the NTG-induced migraine-like attack.

### 4.1. Brain Regions with Altered FC

In this study, a number of regions were observed to be involved following NTG administration over time. Each is discussed below in the context of thalamic involvement/connectivity in the context of the two approaches used.

#### 4.1.1. SCA Approach

*Cortical Regions:* Areas known for their role in pain modulation, such as the insula and the orbitofrontal cortex, showed a progressive reduction in their functional correlation with the right thalamus from baseline to the prodromal phase and from the prodromal to the full-blown phase, while no significant correlations were observed between the thalamus and the pain-encoding areas such as the primary somatosensory cortex. These results are only partially in line with the data from spontaneous migraine attack recorded by Amin et al. [26] who reported a remarkable thalamic involvement in the modulation of the cortex activity during the ictal phase of the migraine attack, mainly involving the superior parietal lobule, insular cortex, primary motor cortex, supplementary motor cortex, orbitofrontal cortex and the primary somatosensory cortex (S1). In this framework, the insula plays an emerging role since many of its putative functions also appear as migraine symptoms. Being a cortical hub for the integration of extero- and intero-ceptive information inputs that can be transposed into higher-level behavioural function, it has already been thought to play a key role in sensory processing [38], but further studies will be required to specifically focus on its different role during ictal and interictal phases.

Furthermore, during the premonitory phase of the NTG-induced headache attack, the present pilot study is in agreement with the functional involvement of the precuneus (part of the DMN, known to play a role in sensory integration) and cuneus (an area of the visual cortex also thought to be involved in multisensory integration and cognitive processing) proposed recently by Karsan et al. [21], (see Supplementary Materials for a detailed list of all the statistically significant areas involved). Indeed, Karsan and colleagues reported functional alterations of the cuneus as primary findings, while in the present pilot study an extremely small but statistically significant cluster came out. These differences might be explained by the different sample size we considered in this pilot study as well as a different algorithm to process data.

*Brainstem:* Pivotal positron emission tomography (PET) studies support the role of the pons in migraine genesis, in particular the dorsal rostral pons area [39], suggesting that the pathophysiology and genesis of migraine attacks is probably not just the result of one single “brainstem generator” [40]. In line with this hypothesis, but differently from Amin et al. [26], the present data support the notion of the fundamental coupling between the pons structures and the thalamus, suggesting how the alteration of the activity among them leads not only to the premonitory phase, but also regulates recovery after the NTG-induced headache attack. Migraine attack generation therefore includes spontaneous oscillations of complex networks involving the brainstem circuitry, the thalamus and the hypothalamus, even though the latter was not confirmed in this population [14].

*Cerebellum:* Growing evidence from structural and functional neuroimaging studies suggests a cerebellar involvement in migraine, in particular of Crus I, II and Vermis VI [41]. Indeed, the cerebellum is an integrator of multiple effector systems including affective processing, pain modulation, as well as sensorimotor processing; it is even suggested that it has a modulating role in pain perception [42], but the extent of its implication in headache, and specifically in migraine, is not fully understood. The results of this study further underline the relationship between the thalamus and different structures of the posterior cerebellum (lobules VII, VIII and IX), which are known for their role in non-motor processes as attentional/executive integration [43,44]. From an anatomical point of view, cerebellar nuclei neurons send divergent, excitatory axonal projections to various thalamic nuclei that include extensive innervation of migraine-related thalamic areas [45]. Through these connections, the cerebellum has been shown to robustly affect thalamocortical network activity [46]. The cerebellar involvement in migraine pathophysiology has also been suggested through the co-activation of the periaqueductal grey and the posterior cerebellum (Crus I and Crus II areas) during trigeminal pain stimulation in patients experiencing a migraine attack [47]. Cerebellar Crus I and Crus II areas are closely linked to the association cortices, especially the prefrontal and posterior parietal cortical areas, and are thought to be engaged in cognitive and emotional representations, showing overlapping activity between aversive and heat pain [42].

#### 4.1.2. WCA Approach

In order to investigate the full-blown phase, where a comprehensive disruption of the FC coherence between the thalamus and the rest of the brain has been reported in spontaneous migraine attacks [48], we implemented the WCA approach and assessed dynamic changes during the NTG-induced headache.

WCA is a method to study the dynamic changes of FC in order to visualise when, and at which frequency, the activity of two regions/networks co-vary and display phase-locked behaviour, therefore suggesting a functional interaction between them [16]. Indeed, thalamocortical communication at rest was found to be not static, but rather dynamic, in particular with the bilateral insulae, prefrontal cortices and dorsal anterior cingulate cortex. These areas are well known to be part of the salience network, which has shown a temporal instability in its activity in migraineurs [5]. In our pilot study, during the prodrome and full-blown phase of the NTG-induced headache attack, the subjects showed profound dynamic changes in the interaction between thalami and the SN, nowadays labelled as the pain matrix [3,49,50]. Interestingly, the WCA scalogram depicts a scenario in which the thalamus appears to be leading the salience network signal, therefore suggesting a possible role of the thalamus as a pacemaker for the pain matrix functional connectivity [51,52]. Moreover, the scalogram clearly shows the absence of phase coherence between the thalamus and the SN during the NTG-induced headache full blown phase, suggesting a severe disruption of the interaction within the pain circuitries.

### 4.2. Overall Interpretation of Imaging Findings

Taken all together, these data suggest that an altered thalamocortical interaction could be the major contributor to the abnormal multimodal sensory processing during a migraine attack. The thalamocortical dysrhythmia model hypothesizes the alteration of the underlying physiological oscillatory interplay of the activity as the mechanism causing the different manifestations present in specific neurological disorders (e.g., neuropathic pain, tinnitus) as well as neuropsychiatric disorders (e.g., depression) [53,54]. In the migraine field, several studies and different techniques, from magnetoencephalography (MEG) to electroencephalography (EEG) and nowadays to fMRI, already suggest the possible role of the thalamocortical dysrhythmia in leading to the dysfunction of multisensory integration consequently causing disturbances in sensory, cognitive, and motor neural processes in migraineurs [11,48,55,56,57].

Our data further support, not only the notion of the pivotal interplay between thalamus and structures, as well as networks, well known for their role in migraine’s processing, but it also highlights their dynamical modulation, disruption and finally restoration throughout the headache ictal experience. Therefore, it is possible to speculate that the thalamocortical temporal correlation of the activity, so fundamentally involved in the symptom manifestation, is progressively changed during the ictal phase of a migraine attack, sustaining the hypothesis that, once migraine is generated, the thalamocortical dysrhythmia leads/influences the experience throughout all the subsequential phases.

Finally, future studies will also be required to better address the importance of the relationship between the pain matrix-salience network and other potential key regions in migraine pathophysiology, not only including the thalamus, but also the cerebellum, the brainstem, the hypothalamus and the insula.

### 4.3. Limitations

Some considerations need to be addressed with respect to the study limitations that include: (1) Numbers of subjects: from a technical point of view, the small number of enrolled subjects limited the choice of the analyses to be performed on current data. However, since the results are in line with literature, we believe that the proposed approach represents a powerful and reliable tool to investigate functional changes in the brain under a migraine attack. (2) Atlas: we used a modified GIF plus SUIT atlas to parcellate the brain in order to select the seed area before performing SCA analysis. The GIF atlas parcellates the brain according to the geodesic propagation concept, which do not exactly match the functional brain organization, which may alter SCA results. This may represent a limit for any fMRI analysis, though, as the optimal strategy for brain parcellation has yet to be identified [58]. (3) Drug effects of NTG on brain blood flow: NTG itself may affect the BOLD signal dynamics, independently of headache occurrence. It is however worth noting that prodromal scans were acquired on average 65 min. following NTG oral administration, and full-blown scans 140 min following NTG. After oral administration, the onset of vasodilatory effects occurs within 1 to 3 min., with a max effect occurring within 5 min; moreover, NTG is primarily eliminated via metabolism in the liver and has a mean half-life of 2.6 min. [59]. Therefore, any direct vasoactive-related perfusion effects of NTG in the human brain should be excluded. It is however worth noting that preclinically, Greco et al. (2011) [60] showed a persistent effect of NTG on the cortex up to 150 min after the systemic administration of NTG to rats. Future work should consider including control subjects for perfusion effects on the BOLD signal, e.g., people who would not develop headache. (4) NTG induced headache attack: a model of migraine. Considering the difficulties of standardizing the scan(s) of all the evolving phases of a spontaneous migraine attack, the use of a migraine model represents both a limitation and the cornerstone of this study because it allowed the creation of a reproducible acquisition scheme. Experimental human and animal models of migraine have already yielded significant insights into brain structures that mediate migraine symptoms and the recurrence of attacks. Among them, the reliability of the NTG model resides in its ability to reproduce headache attacks with features that are reminiscent of the spontaneous migraine attack [61], as highlighted also in Table 3. Given the statistical relevance of this comparison, it is possible to assert that the brain changes observed were paralleled in the natural state even though a direct comparison would be required to fully prove this hypothesis. (5) Absence of a control group: we originally included a small group of healthy controls in our study, but none of them developed a headache, which posed the issue of the most appropriate timing of the follow-up scans and their actual validity. Due to the totally different clinical response in patients with migraine and healthy subjects and the consequent dissimilar timing of the follow-up scan, we felt that the most reasonable and solid approach to interpret the data from the migraine group was the comparison of each migraine patient to his/her baseline.

## 5. Conclusions

These findings reveal that during the NTG-induced headache attack the whole brain FC changes systematically and dynamically, involving areas well known for their roles in pain modulation and migraine-like experience. Alterations in the brain networks, more than in a single brain structures, are implied in the onset and progression of a migraine attack. Indeed: the brainstem pain-modulating circuitry and hypothalamus oscillating activity have a leading role in migraine generation, but then the dynamical alteration within the thalamocortical and cerebellar interaction leads the progression and resolution of the migraine experience. This hypothesis needs to be further confirmed on a larger sample size and with other approaches, such as dynamic causal modelling (DCM), shedding light on the comprehensive causal architecture of migraine experience.

## Figures and Tables

**Table 1:** List of the regions of interests (ROI) and relative details (anatomical location, coordinates, according to the Montreal Neurological Institute coordinate system (MNI), of the centroid and radius of the sphere) added to the customised atlas considered for the seed-based correlation analysis.

ROI Location	MNI Coordinates (x,y,z)	*r* (mm)
Left Dorsal Pons [26]	−8 −24 −32	5
Right Dorsal Pons [26]	8 −24 −32	5
Left PAG [29]	−2 −28 −6	3
Right PAG [29]	4 −28 −6	3
Hypothalamus [30]	0 2 −6	3
Left Spinal Trigeminal Nucleus, Pars Caudalis [13]	4 −40 −55	3
Right Spinal Trigeminal Nucleus, Pars Caudalis [13]	−4 −40 −55	3
Dorsal Raphe Nucleus [31]	0 −28 −12	3

**Table 2:** Demographics of the cohort enrolled. Clinical and demographic characteristics are expressed as means ± SD.

Female, n (total)	2 (5)
Age, years	33.4 ± 7.1
Migraine frequency, n. of days per month	4.4 ± 2.7
Disease onset, age in years	13.2 ± 4.5
Disease duration, years	20.2 ± 7.9
Comorbid tension type headache, n (%)	2 (40%)
Tension type frequency, n. of days per month	1.2 ± 1.0
Comorbid anxiety, n (%)	1 (20%)

**Table 3:** Clinical workflow of migraine subjects who developed NTG-induced headache attack and completed the study.

*Subject ID*	*Anamnestic Migraine Features ˄*	*Presence of Not Specific Headache after NTG Administration* *∫*	*MRI Scan 1, Minutes Post NTG*	*Premonitory Features at Prodromal Scan †*	*MRI Scan 2, Minutes Post Induction*	NRS */ Pain Side at Full Blown «	*Migraine Features during Full Blown Scan ˄*	*Self Reported Resemblance with Typical Migraine Attack ~*	*MRI Scan 3, Minutes at Recovery after NSAID*	NRS * Recovery
*EM1*	0/0/1/1/1/1	1	80	0/0/0/1/0/0/0/0/0/1/0	200	7/R	0/0/1/1/1/1	1	85	1
*EM2*	0/0/1/0/1/1	1	31	1/0/0/1/1/0/0/0/0/0/0	140	5/R	0/0/1/0/1/1	1	50	0
*EM3*	1/1/1/1/0/1	1	110	0/0/0/0/1/0/0/0/1/1/0/0	155	6/L	1/0/1/1/0/1	1	70	1
*EM4*	1/0/1/0/1/1	0	38	1/1/0/0/0/0/0/1/0/0/0/0	70	8/B	1/0/1/0/1/1	1	95	0
*EM5*	0/0/1/1/1/1	0	65	0/0/0/0/1/0/0/1/1/0/0	105	6/B	1/0/1/1/1/1	0	106	1
***Median***			***65***		***140***	6 *			***85***	1 *

˄ 1 = presence of the characteristics of migraine attack: (0 = no; 1 = yes) nausea/vomiting/photophobia/phonophobia/aggravation by movement/throbbing pain. ∫ a specific headache: 1 = presence of transient olocranic headache after NTG administration, not resembling a migraine attack neither fulfilling ICHD-III criteria. † 1 = presence of prodromal signs or symptoms: (0 = no; 1 = yes) yawning/irritability/mood swing/sleepiness/tiredness/loss of appetite/food desire/nausea/stiffness/thirst/urinary retention. * NRS: numeric pain rating score 0–10 (mild: 1–3; moderate: 4–6; severe: 7–10). ~ 1 = self-reported resemblance between her/his typical migraine attack and the NTG-induced headache attack (0 = no; 1 = yes). « R = right; L = left; B = bilateral.

## Data Availability

Data used for this study are available on request to the corresponding author.

## Supplementary Materials

The following are available online at https://www.mdpi.com/2076-3425/11/2/165/s1. The file contains the detailed list of the brain areas statistically significantly connected with the left thalamus and the relative change in FC (*rcFC*) index in each phase, normalised for baseline FC values.
